# Infant and dyadic assessment in early community-based screening for autism spectrum disorder with the PREAUT grid

**DOI:** 10.1371/journal.pone.0188831

**Published:** 2017-12-07

**Authors:** Bertrand Olliac, Graciela Crespin, Marie-Christine Laznik, Oussama Cherif Idrissi El Ganouni, Jean-Louis Sarradet, Colette Bauby, Anne-Marie Dandres, Emeline Ruiz, Claude Bursztejn, Jean Xavier, Bruno Falissard, Nicolas Bodeau, David Cohen, Catherine Saint-Georges

**Affiliations:** 1 Department of Child and Adolescent Psychiatry, AP-HP, Hôpital Pitié-Salpêtrière, Paris, France; 2 Department of Child and Adolescent Psychiatry, Centre Hospitalier Esquirol, Limoges, France; 3 Institut National de la Santé et de la Recherche Médicale Unité Mixte de Recherche 1094, Tropical Neuroepidemiology, Limoges, France; 4 PREAUT Association Programme de Recherche et d’Etudes sur l’Autisme, Paris, France; 5 Department of Child and Adolescent Psychiatry, Association Santé Mentale du 13ème, Paris, France; 6 Simlab, Mohamed VI Polytechnic University, Ben Guerir, Morrocco; 7 PMI Center (Center for Protection of Mother and Infant), Paris, France; 8 Department of Child and Adolescent Psychiatry, Strasbourg University Hospital, Strasbourg, France; 9 ISIR, CNRS, UMR 7222, UMPC, Paris, France; 10 INSERM U669, Université Paris-Sud and Université Paris-Descartes, Paris, France; 11 Association CEREP-PHYMENTIN, Paris, France; Universite de Bretagne Occidentale, FRANCE

## Abstract

**Background:**

The need for early treatment of autism spectrum disorders (ASD) necessitates early screening. Very few tools have been prospectively tested with infants of less than 12 months of age. The PREAUT grid is based on dyadic assessment through interaction and shared emotion and showed good metrics for predicting ASD in very-high-risk infants with West syndrome.

**Methods:**

We assessed the ability of the PREAUT grid to predict ASD in low-risk individuals by prospectively following and screening 12,179 infants with the PREAUT grid at four (PREAUT-4) and nine (PREAUT-9) months of age. A sample of 4,835 toddlers completed the Checklist for Autism in Toddlers (CHAT) at 24 months (CHAT-24) of age. Children who were positive at one screening (N = 100) were proposed a clinical assessment (including the Children Autism Rating Scale, a Developmental Quotient, and an ICD-10-based clinical diagnosis if appropriate) in the third year of life. A randomly selected sample of 1,100 individuals who were negative at all screenings was followed by the PMI team from three to five years of age to identify prospective false negative cases. The clinical outcome was available for 45% (N = 45) of positive children and 52.6% (N = 579) of negative children.

**Results:**

Of the 100 children who screened positive, 45 received a diagnosis at follow-up. Among those receiving a diagnosis, 22 were healthy, 10 were diagnosed with ASD, seven with intellectual disability (ID), and six had another developmental disorder. Thus, 50% of infants positive at one screening subsequently received a neurodevelopmental diagnosis. The PREAUT grid scores were significantly associated with medium and high ASD risk status on the CHAT at 24 months (odds ratio of 12.1 (95%CI: 3.0–36.8), p < 0.001, at four months and 38.1 (95%CI: 3.65–220.3), p < 0.001, at nine months). Sensitivity (Se), specificity, negative predictive values, and positive predictive values (PPVs) for PREAUT at four or nine months, and CHAT at 24 months, were similar [PREAUT-4: Se = 16.0 to 20.6%, PPV = 25.4 to 26.3%; PREAUT-9: Se = 30.5 to 41.2%, PPV = 20.2 to 36.4%; and CHAT-24: Se = 33.9 to 41.5%, PPV = 27.3 to 25.9%]. The repeated use of the screening instruments increased the Se but not PPV estimates [PREAUT and CHAT combined: Se = 67.9 to 77.7%, PPV = 19.0 to 28.0%].

**Conclusions:**

The PREAUT grid can contribute to very early detection of ASD and its combination with the CHAT may improve the early diagnosis of ASD and other neurodevelopmental disorders.

## Introduction

Autism spectrum disorder (ASD) consists of a heterogeneous group of neurodevelopmental disorders [1] that are characterized by disturbances in social relationships and communication, repetitive behaviors and narrow interests, and several degrees of severity [2]. Epidemiological studies have reported a recent increase in prevalence, but this is still a matter of debate. This increase is likely attributable to extrinsic factors, such as improved awareness and recognition of the disease, as well as changes in diagnostic practices and the availability of special services [3, 4] and, in part, expansion of the diagnostic criteria [5, 6]. In 2010, the overall prevalence of ASD among eight-year-old children in the United States was 14.7 per 1,000 or one in 68 [7]. In France, the most recent study, based on the handicap registry, reported an overall prevalence rate of 35/10,000 for ASD among children of the same age group [8].

It is commonly accepted that children with ASD should be enrolled in treatment programs as early as possible [9–14]. The goals of these early interventions are to increase adaptive communication and socialization behaviors, decrease maladaptive behaviors, reduce distress, and increase the quality of life [15, 16]. Some authors argue that very early treatment in infants could inflect the deviant trajectory before installation of the full autistic syndrome and even prevent autism [9]. Indeed, recent studies have attempted to assess the feasibility, effectiveness, and benefits of very early intervention with parents and infants at risk for autism, with encouraging results [17–20]. However, the need for early treatment necessitates the development of tools that allow screening (or the detection of at-risk children) as early as possible [21].

The minimum age for reliable early diagnosis of ASD encompasses several research questions. Although the symptoms of ASD are often present early in life [22–25], the diagnosis of ASD is generally made between the ages of three and five years [24, 26–28]. There are at least five contributing factors that explain this situation: (1) parental concern is not sufficiently taken into account [23, 29–31], (2) the onset of ASD sometimes occurs after the second year of life [32, 33], (3) infants are not developmentally mature enough to meet the diagnostic criteria, (4) differential diagnosis issues are complex at an early age (this is particularly relevant for severe language impairment and intellectual disability, ID), and (5) diagnosis is risky before the age of two years because it is more likely be unstable [34]. The diagnostic criteria of ASD have changed in recent years [35]. Some authors suggest that the present classification system and other factors may contribute to the increasing instability of community-assigned labels of ASD [36]. The initial diagnosis may evolve toward recovery or delayed development without autistic traits. Indeed, early detection cannot be isolated from the possibility of interference with the outcome, as the detected children are more likely to receive support. Some authors suggest that early intervention could diminish autistic symptoms and improve developmental outcomes for a significant proportion of children, and may even be able to reverse the secondary processes of autism [9], or prevent the installation of ASD [17].

Since Baron-Cohen tested the Checklist for Autism in Toddlers (CHAT) on 18 month-old children [37], several studies have attempted to develop screening tools, most often for at-risk toddlers (e.g., children who are evaluated for suspected autism, siblings of such children, and infants with genetic diseases) or toddlers who have already been diagnosed with ASD through clinical judgment and other validated tools (e.g., the Autism Diagnostic Interview, Autism Diagnostic Observation Schedule, and Childhood Autism Rating Scale). These types of studies face several challenges [38]. First, studies with at-risk infants tend to produce much higher positive predictive values (PPVs) than community-based screening studies, because a higher prevalence rate implies a higher probability for a positive result to be correct [39]. In contrast, studies of the general population need to screen a very large sample to obtain enough positive diagnoses in childhood. Many studies cannot assess the sensitivity and specificity of the screening tool because they only assess children who screened positive, which does not capture false negative cases [38]. These studies estimate accuracy with a PPV (calculated from the observed false positive rate) and evaluate sensitivity by calculating the difference between the observed and theoretical prevalence rates. In addition to the accuracy of the tool, early screening is confronted with another issue: the uncertain stability of ASD before the age of two years [40].

Screening tools for toddlers during the second year of life include the CHAT [41, 42] or a modified version of the CHAT, including the M-CHAT (Modified-CHAT)) [43, 44] and the Q-CHAT (Quantitative-CHAT) [45]; the Checklist for Early Signs of Developmental Disorders (CESDD) [46]; the Brief Infant-Toddler Social and Emotional Assessment Questionnaire (BITSEA) [47]; the Young autism and other developmental disorders Checkup Tool at 18 months (Yacht 18) [48], the Social Attention and Communication Study (SACS) [49]; the Early Screening of Autistic Traits Questionnaire (ESAT) [50], and the Social Communication Questionnaire (SCQ) [51]. The CHAT was found to have a good specificity, but low sensitivity [52]; the M-CHAT showed better sensitivity, but produced many false positive scores. More recently, a new two-stage M-CHAT procedure, with follow-up interview, detected potentially indicative psychometric values in a low-risk sample [44]. Researchers are now trying to screen for autism at a very early age.

Few tools have been tested prospectively on infants as young as 12 months. Two have been developed for, or tested on, at-risk siblings. 1) The Autism Observation Scale for Infants (AOSI) is a behavioral observation scale that was shown to predict autism in at-risk siblings who were 12 and 14 months old, but failed to predict the outcome at six and seven months of age [53, 54]. 2) The First Year Inventory (FYI), a parental questionnaire, also showed promising results [55]. Three tools have been assessed in the community. 1) The FYI has been used in a population-based study with a small sample [56, 57]. 2) The SACS is a behavioral inventory that reported striking results for the screening of 12-month-old infants in the community [49], but failed to be confirmed by further research. 3) The Communication and Symbolic Behavior Scales Developmental Profile Infant-Toddler Checklist (CSBS-DP IT-Checklist) is a parental questionnaire that demonstrated high specificity, but low sensitivity and PPV [58, 59].

Only three tools have been prospectively tested and shown to have predictive value for children of less than one year of age. 1) The CSBS-DP IT-Checklist was used to screen the general population at 6 to 8 and 9 to 11 months of age [60] and 2) the Taiwan Birth Cohort Study (TBCS) screened six-month-old infants in the community [61]; both tools showed a low PPV. 3) The PREAUT (Programme de Recherches et d’Etudes sur l’AUTisme) grid was developed to screen very early at-risk infants.

Several authors have proposed a paradigm shift from the assessment of infant behavior to dyadic assessment of interactions, because delays in developmental milestones and impairments in early social interactions are not sufficient to predict ASD. They argue that early screening of ASD should rely on dyadic/interactive behaviors, rather than infant/toddler behaviors. The developmental cascades perspective suggests that dysfunction in one system can influence another system over time to shape the course of development [62]. Recent studies in ASD, using a retrospective approach through home movies [25, 63–65] or a prospective approach with at-risk samples (e.g., siblings [66–69]), provide support for this shift by examining the quality of early interactions through synchrony, reciprocity, and emotional engagement [70]. Indeed, mothers of at-risk infants try to compensate for the lack of interactivity of their child by intensifying their stimulation from very early on [65]. Green proposed that interactive specificities of infants at risk for autism may modify parents’ behavior in interactive cycles [71].

Thus, it appeared necessary to develop a tool that focused on the infant’s spontaneous ability to provoke both behavioral and emotional interactions with its care-giver [70], rather than focusing on a few isolated infant behaviors or general skills. The PREAUT grid was developed for this purpose and was tested at nine months of age on infants who had West syndrome and were at high risk for ASD [72]. Patients who screened positive had a risk of developing ASD or ID at age four. The tool showed a good PPV, but only in a small sample of at-risk infants who had West syndrome [72]. Findings from samples of high-risk children might not be generalizable to the low-risk population [73]. Here, we assessed the ability of the PREAUT grid to predict ASD during the first year of life in the community. We screened the children at 4, 9, and 24 months of age, as infants are systematically examined at these three ages in France. The goal was to implement a feasible screening procedure, beginning as early as possible, that could open the way to preventive care for at-risk children. The M-CHAT revised with follow-up was not validated when our study began. Thus, we used the CHAT for the 24-month screening, as it was the best validated tool at that time. We hypothesized that (1) an early positive PREAUT screen would predict a later positive CHAT screen; (2) an early positive PREAUT screen would predict ASD at three to four years of age; and (3) multiple screens would improve sensitivity and specificity of the detection process.

## Methods

### Design and participants

In this prospective multi-centric study, infants were enrolled in the PMI centers of 10 French departments between September 2005 and November 2011. A pilot study was conducted before 2005 to assess the feasibility of training many PMI (Mother/Infant Protection) physicians to use and score screening tools (PREAUT and CHAT) in their current practice. Infants are systematically examined at 4, 9, and 24 months of age in the French healthcare system. The PMI system was designed to allow all families, including those of low socio-economic status, to access free medical care and prevention. No socio-demographic data were collected. The only inclusion criterion was being a child entering a PMI service. Exclusion criteria were parents’ refusal to consent to the follow-up assessment and/or the research protocol. Parents provided verbal informed consent after they received verbal and written information about the study. The Institutional Review Board (*Comité de Protection des Personnes de l’hôpital de Saint Germain en Laye*) approved the study (December 14^th^, 2000). We screened 12,179 infants with the PREAUT grid at four (PREAUT-4) and/or nine (PREAUT-9) months. Of these, 4,835 toddlers were screened with the CHAT at 24 months (CHAT-24).

### Screening tools

#### PREAUT grid

The PREAUT grid was developed through observation of family home movies of babies who were later diagnosed with autism and clinical work with at-risk infants [74]. Laznik hypothesized that babies who are at risk of developing ASD may present a deficit of the innate need to interact and be a source of pleasure for the person with whom they interact, in contrast to healthy infants [70]. The PREAUT grid evaluates the infant’s ability to spontaneously engage in synchronous and joyful interactions [72]. The PREAUT grid items (*e*.*g*. spontaneously or not looking at the examiner/soliciting his mother) were formulated to reflect the lack of social initiative; the more an infant is actively engaged during an interaction, the higher the score. The grid is scored by a pediatrician during a visit with the infant and its mother (or another care-giver). The doctor observes how the infant behaves, with him and with its mother, not only when it is solicited but also when nobody directly engages it. The grid is provided in S1 Table.

The grid includes an initial group of four items and a second group of six complementary items. The second group of items is only completed if the infant showed at-risk behaviors in response to the first four items (score for the first group of items ≤ 3 at four months or ≤ 5 at nine months). Items are weighted in the grid such that, at four months of age, infants are scored “positive” when they do not spontaneously look at the observer, do not spontaneously elicit the gaze of their mother (or other significant caregiver), and do not try to provoke positive reactions from their mother (or another significant caregiver). At this age, the pathological at-risk threshold was set to ≤ 3 (for a maximum score of 15) based on previous work on West syndrome [72]. In a preliminary exploratory study in the general population, we found that very few infants (one ASD case out of three positive infants) met the pathological threshold at nine months of age with the same threshold. Thus, we decided to move the threshold to ≤ 5 of 15, which appeared to be the best cut-off to define more infants as at-risk without decreasing specificity (with the new threshold, the rate of true positive became four ASD cases out of eleven positive infants). Thus, at nine months of age, infants were scored “positive” when they did not spontaneously elicit the gaze of their caregiver or try to provoke positive reactions from their caregiver, and either did not spontaneously look at the observer/or did not try to be looked at by their mother (vocalizing, intensely gazing, or wriggling) even when she was addressing them. Thus, with this new threshold, the nine-month-old infant was considered to be at-risk if it did not respond to its mother’s attempts to engage it, even if it looked at the observer, whereas the four-month-old infant was considered to be not at-risk, provided that it looked at the observer. We did not calculate interrater reliability in this study. However a previous inter-rater reliability study using the PREAUT grid, with other raters but conducted and supervised by the same team, found Kappa coefficients between 0.74 and 1 for each item [72].

#### The Checklist for Autism in Toddlers (CHAT)

The CHAT is a screening instrument that identifies children who are at risk for autism by assessing common play habits and behaviors for infants between the ages of 18 and 24 months. Nine questions that evaluate social interest, motor play, pretend play, pointing, and showing are addressed to parents (items A), and five items assess the child’s behavior and reactions to stimuli that are initiated by the examiner (items B: gaze exchange, pretend play, proto-declarative pointing, pointing comprehension, and constructing a tower with blocks) [41, 52, 75]. Infants are considered to be at high risk when they fail all five key items. Infants are “positive”, with a medium risk, if they fail to point (proto-declarative pointing), both as reported by the mother (A7) and observed by the examiner (B4) [37]. We set the threshold to medium risk to increase sensitivity. The CHAT was administered at 24 months of age to coincide with the systematic examination schedule in France.

### Procedure

The first step of the study involved training six hundred pediatricians and general practitioners, who worked in the mother/infant protection services (PMI) of the 10 departments, to use the screening tools. Training included a presentation of general information on autism, the study’s goals and methodology, and the study screening tools. Practitioners role-played in small groups to learn how to use the instruments. Video sessions were conducted to illustrate scoring or assess their ability to use the tools.

**Fig 1:**
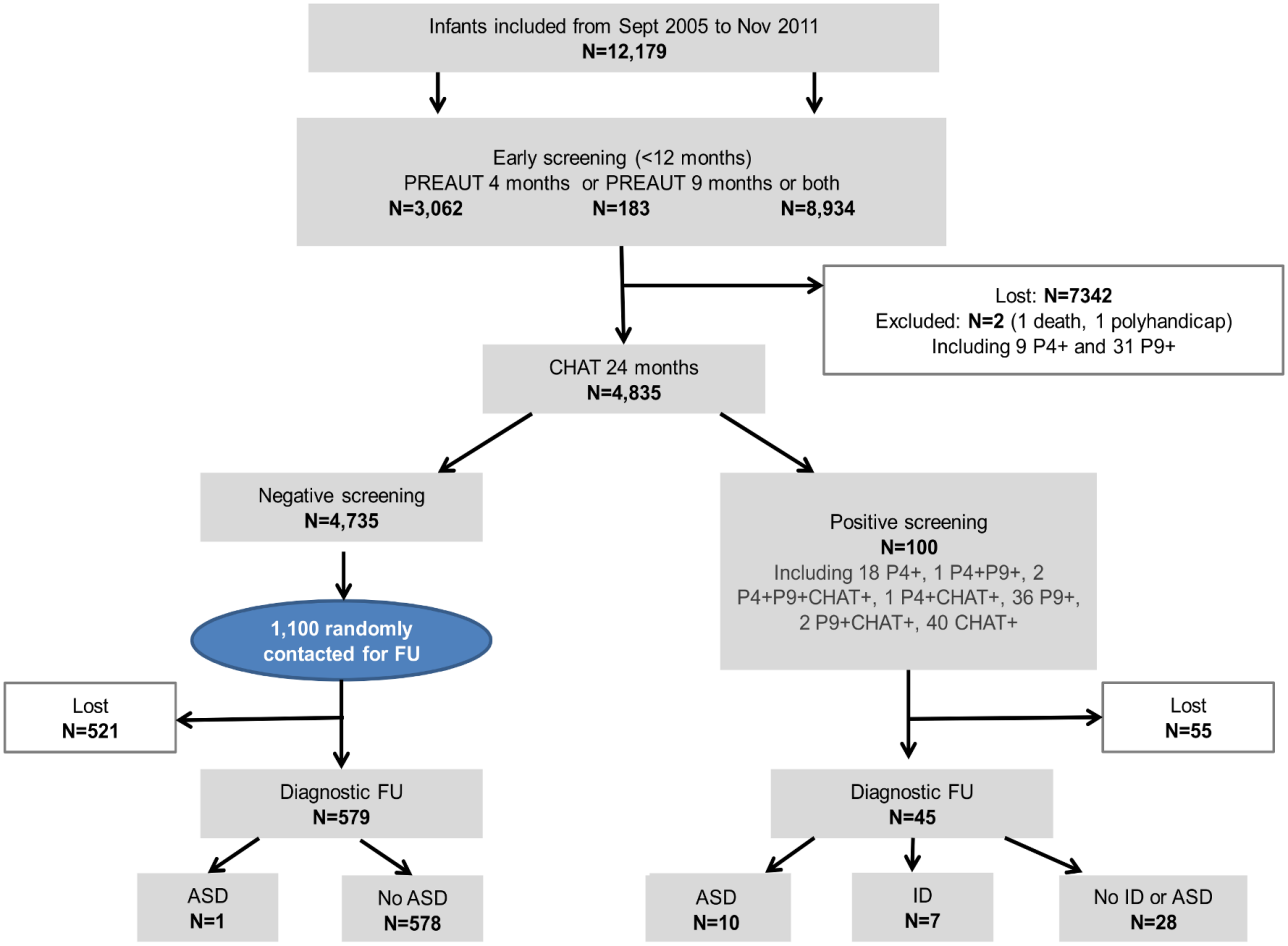
Flow chart.

The second step aimed to screen at least 10,000 infants to obtain sufficient statistical power to account for a drop-out rate of 50% during the two years of screening. The flow of the study is summarized in Fig 1.

During the first year, we screened 12,179 infants with the PREAUT grid at four and/or nine months, of which 8,933 were screened at both ages. Overall, 3,062 infants were screened with the PREAUT grid at four months only and 183 at nine months only, because of missing data or cancelled visits. Of the 12,179 infants screened with the PREAUT grid, 4,835 toddlers were screened with the CHAT at 24 months, indicating that 7,342 were lost during follow-up before the two-year visit. Two patients were excluded due to premature death and poly-handicaps. There were no significant differences between infants screened with the CHAT and those lost by the 24-month assessment for gender (χ^2^ = 0.37, p = 0.54) or age at the first PREAUT grid assessment (mean = 3.82 months (SD = 1.69) vs. mean = 3.79 (SD = 1.68), t = -1.17, p = 0.24). However, there was a significant difference in PREAUT grid scores (mean = 14.46 (SD = 2.05) vs. mean = 14.59 (SD = 2.69), t = 3.17, p = .002). There were no differences in the frequency of individuals who screened positive at the first PREAUT grid assessment (1% vs. 0.8%, χ^2^ = 1.12, p = 0.29).

The third step consisted of (1) the medical diagnosis between three and four years of age for the infants and toddlers who screened positive, using any of the screening tools, and (2) estimating false negatives. All 100 children who screened positive (using either the PREAUT grid at four or nine months, or the CHAT at 24 months) were offered a follow-up appointment. Developmental milestones were systematically assessed during the compulsory visits and included perinatal parameters (pregnancy, childbirth, term birth weight, Apgar) and medical history (for the child and family). Physicians from the PMI center planned physical examinations during the child’s third or fourth year in which a trained psychologist administered the Childhood Autism Rating Scale (CARS) and Wechsler Preschool and Primary Scale of Intelligence (WPPSI) or Brunet-Lezine test. When appropriate, the psychologist clinically assessed ASD symptoms to establish a positive clinical diagnosis according to the ICD-10 classification. In addition to this follow-up appointment, children who were positive at any step of the screening process were immediately referred to specialized care services which subsequently provided clinical assessment for the final diagnosis. In general, French recommendation for autism made compulsory to use a standardized instrument (e.g. Autism Diagnosis Observation Schedule or Autism Diagnostic Interview) [76]. However, given the sample size, these assessments were not conducted by the research team and rather by specialized care services close to patient’s family home. If the diagnosis of ASD was still questionable, a child psychiatrist from the research team obtained supplementary information from handicap services and the child’s school to better understand the educational arrangements for the child. In total, 45 children (45%) received an estimated diagnosis at the end of the study. Of the 17 children receiving an ASD or ID diagnosis, 15 were followed by specialized services professionals who provided a diagnosis and/or described symptoms consistent with the ASD or ID diagnosis; for the other two cases, the parents refused specialized consultations, but the PMI practitioner and school psychologist (based on behavioral observation and psychometric tests) provided a description of symptoms concordant with ASD (in one case) and ID (in the other case). Finally, all diagnoses were based on clinical symptoms according to CIM-10 diagnosis. As regards complementary testing and assessments, of the 7 ASD cases detected by PREAUT screening, 2 were assessed with gold-standard diagnosis tools in a specialized center, 4 had a broader assessment to explore ASD and ID (CARS, WIPPSI, WISC, MRI, EEG, genetic testing) and 1 received only a clinical CIM-10 diagnosis. For the 6 ASD cases detected by the CHAT at 24 months, 2 were assessed in a specialized center, 3 had broader assessment to explore ASD and ID, 1 had only a clinical diagnosis (see Table B in S2 Table). Six children received an “other” diagnosis (see details in the Results section). Information to support the diagnoses came from specialized services for three, from nursery school examinations for two, and from the school psychologist for the last case. For the 22 children who received no diagnosis (healthy children), most simply had a clinical evaluation (with or without tests) that was supported by school feed-back indicating that the child was developing well. There were no significant differences between those who received an estimated diagnosis and those who were lost to the study based on gender (χ^2^ = 0.07, p = 0.79), age at first PREAUT grid assessment (mean = 4.14 months (SD = 1.37) vs. mean = 3.95 (SD = 1.02), t = -0.767, p = 0.44), or the percentage of children at risk after CHAT screening (χ^2^ = 0.50, p = 0.48). However, there was a significant difference in PREAUT grid scores (mean = 8.98 (SD = 6.35) vs. mean = 12.0 (SD = 4.19), t = 2.73, p = 0.008), such that children who were positive by the PREAUT grid screen had better follow-up rates than those who did not screen positive during the first year.

We randomly selected 1,100 children who were negative by all screening instruments to assess their outcomes and identify neurodevelopmental disorders (false negative cases). The physicians from the PMI centers obtained information concerning the children at four to five years of age through systematic reviews that were performed at school or in follow-up appointments. Most had a "nursery school examination" that included general aspects of how they functioned in school and more specific aspects of development, such as gross motor skills, body image, fine motor skills, perceptual organization, language expression, and language comprehension. If the diagnosis was questionable, a child psychiatrist from the research team obtained supplementary information from handicap services. Children were lost for diagnosis when their information was incomplete. A total of 579 children (52.6%) had an estimated diagnosis at the end of the study. Among the 1,100 children who were randomly selected and showed negative results for all screening tools, there were no significant differences between those who received an estimated diagnosis and those who were lost for diagnosis based on gender (χ^2^ = 0.007, p = 0.93), age at first PREAUT assessment (mean = 3.79 (SD = .99) vs. mean = 3.86 (SD = 1.35), t = .99, p = 0.32), or PREAUT score (mean = 14.55 (SD = 1.83) vs. mean = 14.42 (SD = 2.09), t = -1.11, p = 0.27).

### Statistical analysis

All statistical analyses were conducted using the statistical package R, version 2.12.2. The α significance level was set to 0.05 and all statistical tests were two-tailed. Qualitative variables were analyzed with chi-square or Fisher’s exact tests and quantitative variables with Student’s t-tests. Odds ratios and 95% confidence intervals were calculated. PPVs were calculated based on the current data and allowed us to answer the following question: "Given a positive test result, what is the new probability of ASD?"

Most studies use theoretical estimations that are based on known ASD prevalence estimates to calculate sensitivity and specificity. Sensitivity often demonstrates the lowest performance and is defined as the ability of a screening tool to correctly identify (true positives) those patients with the disease (ASD). We examined the 1,100 followed-up individuals who were negative for all screenings to calculate sensitivity and specificity. First, we estimated the number of true positives (for the children followed and the children lost after a positive screening) and false negatives in the total sample (in the randomly selected sample of 1,100 children with a preliminary diagnosis and in the children lost to follow-up). We used two approaches using different PPVs. The first estimation was based on the uncorrected PPV for each tool administered to our sample, independently from other screening tests, to determine the prevalence of ASD in children who screened positive, but were lost to follow-up.

The ASD risk for the children who screened positive and were lost to follow-up was overestimated using this uncorrected method, because most were only positive by one screening tool. The risk for the children who were followed who screened positive by several tools was higher than for those who were positive by only one tool. The second estimation used a corrected PPV that accounted for the possibility of scoring positive by only one of several tools. This second estimation used a corrected PPV considering the specific risk of an autistic outcome for infants who were positive by one tool and negative by the others. Thus, the corrected PPV was extrapolated for each lost case from the PPV of the non-lost children with exactly the same combination of screening results: if the lost child was positive at each step, we applied the (higher) PPV of infants with the same screening profile, if the lost child was positive at only one step, we applied the (lower) PPV of infants positive only at the same step.

Recent studies have shown a gender effect interaction with early screening [77]. We thus used a binomial linear mixed model (LMM) to assess whether gender directly or indirectly affected the early screening prediction. We constructed three LMMs to explain the ASD diagnostic status at four years of age. The first included the PREAUT grid screen at four months, gender, and the interaction of the two explicative variables [glm(formula = Diagnosis at follow-up~PREAUT-4+gender+PREAUT-4*gender, family = "binomial", data = data)]. The two other LMMs were similar, one for the PREAUT grid screen at nine months and the other for the CHAT screen at 24 months.

## Results

### Association between the PREAUT grid and the CHAT (Table 1)

**Table 1:** Significant associations between a positive PREAUT at 4 and 9 months and CHAT items.

**CHAT Item + at 24 months**	CHAT Key item	**PREAUT + at 4 months**		**PREAUT+ at 9 months**	
		odds [95%CI]	Adjusted p value (Holm)	odds [95%CI]	Adjusted p value (Holm)
A1: Rough and tumble play		1.65 [0.04–10.39]	1.00	2.73 [0.53–8.79]	0.46
A2: Social interest		6.38 [0.71–27.07]	0.46	5.13 [0.99–16.79]	0.36
A3: Motor development		17.17 [3.14–61.54]	0.03*	8.99 [1.71–30.3]	0.14
A4: Social play		3.88 [0.43–16.25]	0.64	4.26 [1.08–12.16]	0.31
A5: Pretend play	X	11.44 [3.61–31.32]	0.00*	3.33 [0.85–9.46]	0.46
A6: Protoimperative pointing		5.4 [0.6–22.8]	0.49	6.03 [1.52–17.38]	0.14
A7: Protodeclarative pointing	X	8.83 [1.64–30.87]	0.11	8.28 [2.47–22]	0.02*
A8: Functional play		7.02 [1.31–24.4]	0.15	5.05 [1.28–14.48]	0.20
A9: Showing		7.5 [1.82–23.21]	0.06	5.14 [1.55–13.47]	0.11
B1: Eye contact		0 [0–26.5]	1.00	3.37 [0.08–21.24]	0.53
B2: Following a point	X	7.46 [1.39–25.96]	0.15	6.78 [2.03–17.9]	0.05*
B3: Pretend play	X	7.38 [2.66–18.99]	0.00*	3.71 [1.54–8.05]	0.06*
B4: Protodeclarative pointing	X	6.03 [2.26–15.34]	0.01*	2.18 [0.86–4.85]	0.46
B5: Tower of blocks		5.19 [1.65–14.07]	0.06	2.35 [0.8–5.71]	0.46
**CHAT medium and high risk at 24 months**		**PREAUT**^**+**^ **at 4 months**		**PREAUT**^**+**^ **at 9 months**	
		odds [95% CI]	p value	odds [95%CI]	p value
CHAT medium risk (A7, B4)		18.01 [3.29–64.79]	0.00	12.31 [3.04–36.76]	0.00
CHAT high risk (A5, A7, B2, B3, B4)		78.02 [7.27–469.79]	0.00	38.09 [3.65–220.33]	0.00

Overall, 4,835 children (2,385 girls (49%); 2,450 boys (51%)) were assessed with the PREAUT grid at four and/or nine months and the CHAT at 24 months. One hundred infants were positive on at least one screen and six were positive on two or three tests. We examined significant associations between a positive score on the PREAUT grid and each item of the CHAT using the Holm-Bonferroni adjusted p value (due to multiple analyses). At the age of four months, the PREAUT grid (threshold = 3) significantly predicted failure on several CHAT items at 24 months (A5, B2, B3, B4). Additionally, at nine months, the PREAUT grid (threshold = 5) significantly predicted failure on items A7 (protodeclarative pointing) and B2 (following a point) of the CHAT. Of note, A5, A7, B2, B3, and B4 are the five key items of the CHAT. Moreover, a positive score at four or nine months with the PREAUT grid predicted medium- and high-risk status on the CHAT at 24 months with odds ratios ranging from 12.3 to 78.0 (all p < 0.001) (see Table 1).

### Positive predictive values

Children who were positive by one of the screening instruments (PREAUT-4, PREAUT-9, or CHAT-24) were systematically evaluated to identify developmental disorders (DD), including ID and ASD. Of the 100 children who screened positive at step one, 45 received a preliminary diagnosis at follow-up; of these, 22 were healthy, 10 were diagnosed with ASD, seven with ID, and six with another DD [specific DD of speech and language (N = 2), multidimensionally impaired (N = 2), attention deficit hyperactivity disorder (ADHD, N = 1), and mixed disorders of conduct and emotions (N = 1)]. The clinical outcome of positive infants for ASD and/or ID at one (or more) screening point is shown in Table A in S2 Table. Also, detailed clinical characteristics are given in Table B in S2 Table for children who received a diagnosis of ASD and/or ID. They were 4 females and 13 males. Cases show a large heterogeneity. Interestingly all females were positive at early screening and received a diagnosis of ID comorbid or not with ASD including 2 with a causal genetic condition. In terms of timing of screening new patients whom diagnosis was confirmed at follow-up, we had 10 individuals (5 with ASD and 5 with ID) who were positive at 4 months screening, 3 new individuals (2 with ASD and 1 with ID) who were positive at 9 months screening, and 4 additional individuals (3 with ASD and 1 with ID) who were positive at 24 months screening, leading to a total of 17 individuals with ASD or ID.

The estimated PPVs are presented for ASD only and for neurodevelopmental disorders (ASD or ID) in Table 2. Details on the estimations for the total sample of 4,835 individuals are provided in S3 Table for ASD only and S4 Table for ASD and ID. For the PREAUT-4, the mean PPV was 25.9% for ASD and 52.2% for global neurodevelopmental disorders (ID+ASD). For the PREAUT-9, the mean PPV was 28.3% for ASD and 39.2% for ID+ASD. For the CHAT-24, the PPV was 26.6% for ASD and 32.7% for ID+ASD.

**Table 2:** Estimations of PPV, NPV, specificity, and sensitivity for ASD (top) and neurodevelopmental disorders with each screening tool.

	***AUTISM SPECTRUM DISORDER***								
	Screened individuals	True positives	False positives	False negatives	True negatives	PPV (%)	NPV (%)	Specificity (%)	Sensitivity (%)
P4	4,755	5.60–5.79	16.21–16.40	21.58–30.39	4,702.61–4,711.42	25.4–26.3	99.3–- 99.5	99.6–99.6	16.0–20.6
P9	4,530	8.29–14.92	26.08–32.71	18.89–21.26	4,467.74–4,470.11	20.2–36.4	99.5–99.6	99.3–99.4	30.5–41.2
C24	4,835	9.84–12.28	32.72–35.16	17.34–23.90	4,766.1–4,772.66	25.9–27.3	99.5–99.6	99.3–99.3	33.9–41.5
All together P4 or P9 or C24*	4,835	19–28	72–81	8–9	4,726–4,727	19.0–28.0	99.8–99.8	98.3–98.5	67.9–77.7
	***NEURODEVELOPMENTAL DISORDER***								
P4	4,755	11.40–11.58	10.42–10.60	28.78–37.60	4,695.40–4,704.22	51.8–52.6	99.2–99.4	99.8–99.8	23.5–28.4
P9	4,530	13.58–18.62	22.38–27.42	26.60–30.56	4,458.44–4,462.40	33.12–45.4	99.3–99.4	99.4–99.5	33.8–37.9
C24	4,835	13.10–16.37	28.63–31.89	27.07–32.81	4,757.19–4,762.93	29.12–36.4	99.3–99.4	99.3–99.4	33.3–33.6
All together P4 or P9 or C24*	4,835	32–41	59–68	8–9	4,726–4,727	32–41	99.8–99.8	98.6–98.8	79.6–83.4

* Some individuals were positive by several tools. The number of Individuals was rounded to the nearest integer. P4: PREAUT at 4 months, P9: PREAUT at 9 months, C24: CHAT at 24 months

### Sensitivity, specificity, and negative predictive value (NPV)

We first calculated the false negative and true positive cases (S3 and S4 Tables) to calculate sensitivity and specificity. We selected 1,100 toddlers who screened negative at random to determine their outcomes and track false negative cases. Of the 1,100 children, five had not received the CHAT and were excluded, and 516 were lost to follow-up (did not attend the PMI assessment, moved, or refused). Of the remaining 579 children, one female was diagnosed with ASD (see Table B in S2 Table). Of the remaining 578 children without ASD, 52 were diagnosed with other disorders based on the pediatrician’s estimate (language delays, global developmental delays, or conduct disorder). From the 4,735 cases that were negative at all screenings (PREAUT-4, PREAUT-9, and CHAT-24), we extrapolated the number of false negatives from the subsample of 1,100 children who were randomly selected for follow-up. From the 579 cases that had all-negative screens and were followed-up, one had an ASD diagnosis (false negative). Thus, we extrapolated eight false negative cases from the 4,735 cases with negative screens.

We calculated the number of true positive cases using two strategies to account (or not) for the likelihood that screening positive on one tool increased the probability of screening positive on another. Of the 45 children who screened positive on the PREAUT or CHAT and were followed-up, 10 were diagnosed with ASD. Fifty-five children who screened positive on one tool were lost to follow-up (N = 3 PREAUT-4^+^, N = 29 PREAUT-9^+^, N = 1 PREAUT-9/CHAT-24^+^, N = 22 CHAT-24^+^).

We estimated that 18 ASD cases were detected but lost, using the first method, which showed the PPVs for each tool (0.79 at PREAUT-4, 10.92 at PREAUT-9, and 6.28 at CHAT-24, see S3 Table). Thus, the number of ASD diagnoses in our sample of 4,835 toddlers was 36 using this estimation (10 cases that were screened and followed-up, plus 18 true positive cases that were lost to follow-up, plus 8 false negative cases), giving a prevalence of ASD of 0.74% (36/4,835).

The second method provides a lower estimate of true positive cases and accounts for the likelihood that screening positive on more than one tool increases the probability of an ASD diagnosis. We had to simultaneously account for the three screening results to correctly estimate risk. Thus, the risk was lower in our sample of children who were lost to follow-up (with only one child positive on two tools: P9 and C24) than the raw PPV observed in our sample of 45 children who were followed (with many children positive on two or three tools): the PPV of being positive only on the PREAUT-4 decreased from 26.3 to 20.0%, only on the PREAUT-9 from 36.4 to 14.3%, and only on the CHAT-24 from 27.3 to 16.7%. Thus, we estimated that we had nine ASD diagnoses that were detected, but lost (0.60 at PREAUT-4, 4.29 at PREAUT-9, and 3.8 at CHAT-24). The number of ASD diagnoses in our sample of 4,835 toddlers was 27 using this estimation (10 cases screened and followed-up, plus nine true positive cases that were lost to follow-up, plus nine false negative cases), giving a prevalence of ASD of 0.56% (27/4,835).

Table 2 summarizes the NPVs and the sensitivity and specificity for each tool and combined tools for ASD only and ASD plus ID. All specificities and NPVs were above 98%. The mean sensitivity was approximately 18% at four months (PREAUT), 36% at nine months (PREAUT), and 38% at 24 months (CHAT). Notably, repeating the screening at four and nine months with the PREAUT grid detected half of the individuals with a confirmed diagnosis of ASD at follow-up. Furthermore, the mean sensitivity for the combination of the three tools increased substantially, reaching 73% for detecting ASD cases.

We performed the same analysis to estimate the sensitivity and specificity for each tool to detect neurodevelopmental disorders, combining the ASD and ID diagnoses (see Table 2). The estimated sensitivity for each tool ranged from 26 to 36%. The mean sensitivity of repeating the assessment reached 81%. Again, all specificities and NPVs were above 98%.

### Effect of gender

Recent studies have shown a gender effect interaction with early screening [77]. We thus also used binomial linear mixed models (LMMs) to assess whether gender directly or indirectly affected early screening predictions. In each model, we tested whether a diagnosis of ASD at follow-up could be predicted by a specific positive screening result (PREAUT-4, PREAUT-9, or CHAT-24) and gender, and also whether a diagnosis of ASD at follow-up could be predicted by the interaction between a specific screening tool and gender. The PREAT-4 was significantly associated with a diagnosis of ASD at follow-up (β = 3.61; p < 0.001), but gender alone (β = 1.06; p = 0.14) or in interaction with the PREAT-4 was not (β = -1.22; p = 0.42). Results were similar for the PREAUT-9 [PREAUT-9: β = 3.94; p = 0.024; gender: β = .92; p < 0.001; PREAUT-9*gender: β = 0.45; p = 0.78] and the CHAT-24 [CHAT-24: β = 3.69; p < 0.001; gender: β = 0.58; p = 0.42; CHAT-24*gender: β = 0.38; p = 0.80]. In summary, there was no significant effect of gender.

## Discussion

### Summary of the findings

The purpose of this study was to examine the ability of the PREAUT grid to detect ASD at a very early stage of development in the general population. We prospectively recruited more than 12,000 infants, and more than 4,000 were followed-up to 24 months. The outcome of positive infants and a subsample of negative infants was assessed at the age of three to four years. The PPV could be directly calculated, but an extrapolation was necessary to estimate the sensitivity and specificity, due to lost and non-followed up children (see limitations). The PREAUT grid could identify some early risk of autism and other neurodevelopmental disorders in this large sample. The PREAUT grid status at four or nine months was significantly associated with the CHAT status at 24 months. The sensitivity and PPV for the two screening tools were quite similar (sensitivity of approximately 30%, PPV of approximately 25%), even though screening with the PREAUT grid was performed 15 to 20 months earlier than with the CHAT. Notably, the repeated use of the screening instruments and/or their association increased sensitivity to above 70%. Thus, 2/3 of the ASD cases were detected at 24 months of age. Repeating the PREAUT screening at four and nine months detected half of the ASD cases.

We have already shown the ability of the PREAUT grid to predict ASD during the first year of life in a previous study on high-risk infants with West syndrome [72]. In this study, the PREAUT grid was able to correctly detect children with a clinical diagnosis of ASD in a notable proportion (one in four), although it was administered to the general population, in which the prevalence of ASD is relatively low [78]. Moreover, many individuals with false positive results received another developmental diagnosis, with ID being the most frequent. Thus, this proportion increased to one in two for individuals with a global neurodevelopmental disorder (ASD + ID), meaning that one half of the four-month-old infants screening positive were later diagnosed with ID or ASD.

These neurodevelopmental outcomes may justify very early intervention. Jones and Johnson, arguing that there is substantial variability in early developmental trajectories, proposed that early intervention should target “*neurodevelopmental mechanisms that produce troubling symptoms in early development*”, without waiting for clinical diagnosis. It may “*in the long-term ameliorate or even prevent the emergence of troubling symptoms*” [79]. Finally, the PREAUT grid demonstrated its usefulness with trained professionals from child protection services. Thus, this tool may be useful for developing strategies for the early detection of ASD or other neurodevelopmental disorders and initiating care as early as possible, which is crucial for better outcomes. Infants who score positive on the PREAUT grid should be further examined, carefully followed-up, and provided with care to foster the development of interactive abilities. In recent years, several authors have tried to evaluate the benefits of very early intervention for infants at-risk for autism (see for a review Bradshaw, et al., 2015 [19]). For example, a blind randomized trial with 7 to10-month-old at-risk infants suggested that 6 to 12 home-based intervention sessions with parents increased infant attentiveness to parents, reduced autism-risk behaviors, and improved attention disengagement [18]. Another study suggested that early parent-mediated intervention through 10 weekly in-home visits had “the potential to impact the brain systems underpinning social attention in infants at familial risk for ASD” [20]. These latter studies were conducted with non-symptomatic siblings, whose risk for autism is estimated to be up to 20%. In our study, four or nine-month-old PREAUT-positive infants were found to have a risk of autism above 20%. It seems reasonable, therefore, to propose very early detection with intervention for these detected at-risk infants. Moreover, it is possible that early intervention, aiming to intensify parental responsivity to infant cues, may be able to prevent or alleviate, not only the ASD outcome, but also other neurodevelopmental disorders that we found to be associated with a positive PREAUT status.

### Comparisons to other tools and studies

Given the number of studies on ASD screening, we limited our comparison to prospective studies that assessed screening tools during the first two years of life in a community sample. Thus, we excluded retrospective studies (e.g., Q-CHAT: [45], studies on a sample with a mean age ≥ 24 months [46], and many studies on selected populations (pre-screened samples, at-risk samples, diagnosed patients, etc.). Results from research conducted on community samples (including PPV, sensitivity, and specificity when available) are summarized in Table 3.

**Table 3:** Screenings in the first 2 years of life in community-based samples: Prospective studies with available psychometric data.

			Age of screening in months (mean)	Sample size (N)	PPV for ASD (%) (bc = best cutoff)	Estimated Se (%)	Estimated Sp (%)
**PREAUT**	Current study		4	4,755	25.4–26.3	16.0–20.6	99.6–99.6
			9	4,530	20.2–36.4	30.5–41.2	99.3–99.4
CHAT			24	4,835	25.9–27.3	33.9–41.5	99.3–99.3
PREAUT and CHAT			4/9/24	4,835	19.0–28.0	67.9–77.7	98.3–98.5
**CSBS-DP ITC**	(Pierce, et al., 2011)	[58]	12	10,479	17	-	-
	(Wetherby, et al., 2008)	[60]	6-8/9-11/12-14	100/259/330	7/13/7	20/77/91	-
**FYI**	(Turner-Brown, et al., 2013)	[56]	12	699	31 (bc)	<44 (bc)	
**TBCS**	(Lung, et al., 2011)	[61]	6	1,783	19 (bc)	-	-
	"	"	18	1,618	21 (bc)	-	-
**SACS**	(Barbaro & Dissanayake, 2010)	[49]	12/18/24	20,770	90/82/80	Total Se = 83.8	99.8
**ESAT**	(Dietz, et al., 2006)	[50]	14–30 (m = 16)	31,724	24	-	-
**CESDD**	(Mieke Dereu, et al., 2010)	[86]	3–39 (m = 16)	6808	7	80	94
**CHAT** 2-stage	(Gillian Baird, et al., 2000)	[52]	18	16.235	59	21	99.
1-stage	"	"	18	"	8	35	97.7
1-stage	(VanDenHeuvel, Fitzgerald, Greiner, & Perry, 2007)	[87]	18	2,117	58	-	
**M-CHAT** /F	(Kleinman, et al., 2008)	[43]	16–30 (m = 20)	3,309	65	-	-
/F	(Robins, 2008)	[88]	16–27 (m = 21)	4,799	57 (outcome 24 mo)	-	-
/F	(Pandey, et al., 2008)	[89]	16–23 (m = 18)	4,265	28	-	-
/F	(Chlebowski, Robins, Barton, & Fein, 2013)	[90]	16–30 (m = 20)	18,989	54	-	-
/F	(Windham, et al., 2014)	[91]	16–30	1,760	26	-	-
/F	(Robins, et al., 2014)	[44]	16–31 (m = 21)	16,071	47.5 (bc)	85	99
modified	(Kamio, et al., 2014)	[92]	18	1,851	45.5	47.6	98.6
	(Stenberg, et al., 2014)	[93]	18	52,026	1.5	34	93%

The most widely studied tools for screening in the second year of life are the CHAT and M-CHAT. The PPV for the CHAT was lower (27%) in our study than previously reported [52], whereas the sensitivity and specificity were in the same range. This may due to that fact that we administered the test only one time to make the study more feasible and increase sensitivity. Indeed, in the original study, the PPV for the CHAT was 59% when administered two times, but decreased to 8% when administered only once, whereas the sensitivity increased from 21 to 35% [52]. It is reasonable to expect a better PPV for the CHAT in our study than that reported by Baird *et al*. for toddlers screened at 18 months (also only administered once), as the children in our study were 24 months old. The M-CHAT may have a better sensitivity than the CHAT [43, 44], but it was not assessed in 2005 when we started the study. However, combined screening with the PREAUT grid in the first year and the M-CHAT at 18 or 24 months, should be more sensitive than combined screening with the PREAUT grid and the CHAT and should be considered for systematic screening programs in the community.

Prospective screening tools for children of 12 months of age or younger that were tested in the community include the FYI [56, 57], SACS [49], CSBS-DP IT [58, 59], and TBCS [61]. The SACS showed high PPV, sensitivity, and specificity at 12 months [49], whereas the PPV for the other instruments ranged from 7 to 31%. The sensitivity and specificity of the PREAUT grid (which are not always available for other screening tools, see Table 3) were quite comparable to those of other tools, even though it was administered earlier. The specificity was above 98%, and the sensitivity when repeated at four and nine months was above 50%. Adding the CHAT at 24 months to the PREAUT grid at four and nine months increased the sensitivity to 73%. This result confirms that implementing early detection programs, using a two-stage screening approach, could be clinically relevant and lead to earlier detection of ASD [80].

### Implications for early screening of ASD

Our results support a new screening and diagnosis strategy to detect children with potential neurodevelopmental disorders. First, we believe that early screening should, at best, highlight the risk of developing neurodevelopmental disorders, including ASD. Screening tests should be widely administered at an early age. They may not provide a definitive diagnosis, but could indicate a possible developmental disorder that may be too early to define. It is necessary to follow children until they are three or four years old with a more detailed assessment to confirm or rule out the initial diagnosis [48]. However, the recognition of the early warning signs of neurodevelopmental disorders should lead to more detailed and specialized assessment of interactions, and may justify early supportive care without waiting for the final diagnosis.

Second, our findings and those of others suggest that a dyadic approach of interaction that considers both synchrony and emotion may be helpful for assessing the early risk of ASD (during the first year of life) and highlight the importance of simultaneously considering both proclivities rather than considering them to be mutually exclusive [65, 81, 82]. Parents may be excellent informants of pathological processes that are occurring in their developing child [22, 23, 29, 30, 83].

Third, our findings support the view that repeated screening may be the best strategy for increasing sensitivity, which is often the metric with the lowest value in the field of early diagnosis of ASD (see Table 3). Instruments should be adapted to developmental abilities and first-year instruments should not be the same as second-year instruments. First-year instruments that include a dyadic assessment may have added value.

### Limitations

Our study had several limitations. First, our study was affected by considerable sample loss. We lost approximately one half of the individuals at each step of the study. From the 12,179 infants screened with PREAUT grid, only the 100 positive cases of the 4,835 who attended the 24-month consultation, and had the CHAT, were tracked for follow-up. Among these 100 positive cases, only 45 could be effectively followed-up. Thus, PPVs were calculated on only a part of the positive sample. The fact that PMI centers also recruit families with a low educational and socio-economic profile is likely to have contributed to the high number of lost families at follow-up. Notwithstanding, it is important to implement research in real life situations and we need to develop screening strategies that can equally benefit the population that does not always have access to private care. We cannot exclude a possible bias or a random difference between the followed and lost samples. However, there were few lost children (less than 14%) among the individuals who were positive on the PREAUT grid at four months and tracked for follow-up after the CHAT. Thus, the PPV of the PREAUT grid administered at four months is quite robust. In contrast, due to a high rate of loss, the PPV of the PREAUT grid administered at nine months and the CHAT at 24 are an estimate based on the hypothesis that the lost sample was similar to that which was followed-up. Estimation of sensitivity and specificity required identifying the false negative cases. We used a randomly selected subsample of negative infants and assumed that the sample of negative infants tracked but lost was similar to that which was tracked and actually followed-up. Two factors support this assumption: 1) comparison of the data from followed-up and lost individuals showed no significant differences in gender or age at first assessment; 2) the prevalence of ASD (0.56 to 0.74%) based on these estimates was concordant with the expected prevalence (0.67%) based on epidemiological studies [76].

Second, ASD was not always assessed using gold standard diagnosis tools (e.g., ADI-R [84] or ADOS [85]). We could not always organize direct assessments due to the large geographical distribution of the more than 10 French districts involved in the study. However, the use of information from psychiatric care teams and, in some cases school psychologists, provided a detailed description of child symptoms and functional impairments, leading to a carefully argued CIM-10 diagnosis. Also, false negative cases were generally poorly assessed for ID because the study focused on autism, and the possibility of an ID diagnosis in the negative sample was not carefully investigated.

Third, we could not evaluate the effect of the evaluations on child outcomes through early intervention, as this was not systematically monitored. However, within the context of developmental cascades [62], it is likely that clinical follow-up and support at this early age may have influenced the course of development. Finally, the screening test at 24 months was the CHAT, which is known to be less sensitive than the M-CHAT. However, the M-CHAT and its metric values were not available when our study began.

Further studies should repeat and confirm these results, in the community or in other at-risk populations (such as siblings), using the M-CHAT at 18 or 24 months. They should also better follow the effect of the received intervention on the outcome and propose more standardized assessment tools (such as ADI-R and ADOS). The loss of children to follow-up could also be minimized if disability services registration were available in France for research purposes, as it is in some other countries. Such registries are currently available in only two districts in France [78].

## Conclusion

The purpose of this study was to examine the validity of the PREAUT grid as a screening instrument to detect infants with ASD from a general population sample. Observed PPV, and estimated sensitivity and specificity support the use of the PREAUT grid for very early screening of ASD and other developmental disorders in the community, making it possible to identify infants and families requiring support early, thus reducing the impact of the autism or ID outcome. Repeating the use of screening instruments, with different approaches, increased the sensitivity of the screening process while maintaining the PPV, NPV, and specificity values. Our results also indicate that dyadic assessment (synchrony and emotion) can help to detect ASD risk at very early stages child of development. Healthcare providers are essential for detecting developmental problems earlier, including ASD, by regular monitoring of development, so that children can access interventions earlier.

## Supporting information

S1 Table PREAUT grid. (DOCX)

S2 Table **Table A. Clinical outcome of positive infants at one or more screening points. Table B. Clinical characteristics of ASD and ID cases detected (17 true positive cases) or missed at follow-up (1 false negative case).** ASD: autism spectrum disorder; PDD-NS: pervasive developmental disorder; ID: intellectual disability; ADHD: attention deficit/hyperactivity disorder; WISC: Wechsler intelligence scale for children; WIPPSI: Wechsler preschool and primary scale of intelligence; EEG: electro-encephalogram; MRI: resonance magnetic imaging; CARS: children autism rating scale; ADOS: autism observation schedule; ADI: autism diagnostic interview; VABS: Vineland adaptative behavior scale; ECA-R: *echelle des comportements autistiques révisé*es [scale for autistic behaviors].
(DOCX)

S3 Table Estimation of ASD diagnosis status for (1) infants positive at one screening but lost at FU; (2) infants negative at all screenings and estimated through the random sample. * Some individuals were positive at several tools. Number of Individuals are rounded to nearest integer.
(DOCX)

S4 Table Estimation of ND (ASD or ID) diagnosis status for (1) infants positive at one screening but lost at FU; (2) infants negative at all screenings and estimated through the random sample. * Some individuals were positive at several tools. Number of Individuals are rounded to nearest integer.
(DOCX)
