# Supplementary material for: Infant and dyadic assessment in early community-based screening for autism spectrum disorder with the PREAUT grid
Source: PLoS One. 2017 Dec 7;12(12):e0188831. doi: 10.1371/journal.pone.0188831 (PMC5720624; doi:10.1371/journal.pone.0188831)
Supplement: S1 Table — (DOCX) [file pone.0188831.s001.docx]

**S1 Table:** PREAUT grid

| **4th to 9th month: 1st part of questionnaire** | | |
| --- | --- | --- |
| QUESTION | RESPONSE | VALUE |
| **1. Does the baby seek to look at you?** |  |  |
| a) Spontaneously | Yes | 4 |
|  | No | 0 |
| b) When you speak to him (proto-conversation) | Yes | 1 |
|  | No | 0 |
| 2**) Does the baby seek to have his mother’s (or her substitute) attention?** |  |  |
| a) Without any solicitation from her by making noise, or moving while staring at her | Yes | 8 |
|  | No | 0 |
| b) When she speaks to him (proto-conversation) | Yes | 2 |
|  | No | 0 |
| TOTAL SCORE |  |  |
| If the score is greater than 3 at 4 months or greater than 5 at 9 months, do not reply to questions 3 and 4 |  |  |
| **4th to 9th month: 2nd part of questionnaire** |  |  |
| QUESTION | RESPONSE | VALUE |
| **3) Without any stimulation by his mother (or her substitute**) |  |  |
| a) Does he look at his mother (or her substitute) | Yes | 1 |
|  | No | 0 |
| b) Does he smile at his mother (or her substitute) | Yes | 2 |
|  | No | 0 |
| c) Does the baby try to have an exciting exchange with his mother (or her substitute), for example by giving her or extending his toes or his hand to be kissed or sucked? | Yes | 4 |
|  | No | 0 |
| **4) Following stimulation by his mother (or her substitute**) |  |  |
| a) Does he look at his mother (or her substitute) | Yes | 1 |
|  | No | 0 |
| b) Does he smile at his mother (or her substitute) | Yes | 2 |
|  | No | 0 |
| c) Does the baby try to have an exciting exchange with his mother (or her substitute), for example by giving her or extending his toes or his hand to be kissed or sucked? | Yes | 4 |
|  | No | 0 |
| TOTAL SCORE |  |  |
