# Supplementary material for: Infant and dyadic assessment in early community-based screening for autism spectrum disorder with the PREAUT grid
Source: PLoS One. 2017 Dec 7;12(12):e0188831. doi: 10.1371/journal.pone.0188831 (PMC5720624; doi:10.1371/journal.pone.0188831)
Supplement: S2 Table — Table A. Clinical outcome of positive infants at one or more screening points. Table B. Clinical characteristics of ASD and ID cases detected (17 true positive cases) or missed at follow-up (1 false negative case). ASD: autism spectrum disorder; PDD-NS: pervasive developmental disorder; ID: intellectual disability; ADHD: attention deficit/hyperactivity disorder; WISC: Wechsler intelligence scale for children; WIPPSI: Wechsler preschool and primary scale of intelligence; EEG: electro-encephalogram; MRI: resonance magnetic imaging; CARS: children autism rating scale; ADOS: autism observation schedule; ADI: autism diagnostic interview; VABS: Vineland adaptative behavior scale; ECA-R: echelle des comportements autistiques révisées [scale for autistic behaviors]. (DOCX) [file pone.0188831.s002.docx]

**S2 Table.**

**Table A. Clinical outcome of positive infants at one or more screening points**

|  | **Positive screening** | **Available at FU** | **ASD at FU** | **ID at FU** | **Other disorder at FU** | **Healthy at FU** |
| --- | --- | --- | --- | --- | --- | --- |
| P4 only | 18 | 15 | **3** | 4 | 0 | 8 |
| P9 only | 36 | 7 | **1** | 1 | 2 | 3 |
| C24 only | 40 | 18 | **3** | 1 | 4 | 10 |
| P4+P9 | 1 | 1 | **0** | 0 | 0 | 1 |
| P4+C24 | 1 | 1 | **0** | 1 | 0 | 0 |
| P9+C24 | 2 | 1 | **1** | 0 | 0 | 0 |
| P4+P9+C24 | 2 | 2 | **2** | 0 | 0 | 0 |
| Total | 100 | 45 | **10** | 7 | 6 | 22 |
| P4: Preaut grid at 4 months; P9: Preaut grid at 9 months; C24: CHAT at 24 months; FU: Follow-up | | | | | | |

**Table B. Clinical characteristics of ASD and ID cases detected (17 True Positive Cases) or missed at follow-up (1 False Negative case).**

|  | **Gender** | **P4** | **P9** | **C24** | **CIM-10 Diagnosis** | **Available assessment** | **Clinical charateristics** | **Last educational arrangement in 2015 (age 4-9 year)** |
| --- | --- | --- | --- | --- | --- | --- | --- | --- |
| **14202** | F | + | + | + | ASD  (PDD-NS +ID) | VABS, ADOS-ADI, Brunet-Lézine | Language, motor and cognitive delay, social withdrawal and loneliness, emotional dysfunction | Special education clinics |
| **15639** | M | + | + | + | ASD  (PDD-NS +ID) | WISC, genetic investigation, MRI, EEG | Language delay (no use of “I”), motor stereotypies, intolerance to change, immutability, mild ID | Special class within regular school + ambulatory rehabilitation |
| **5217** | M | + | - | - | ASD  (PDD-NS) | CARS, WIPPSI | Language delay (no use of “I”, receptive language deficit), tantrums despite social inhibition and withdrawal | 4th grade in a regular school but a special education class is to be found in the near future |
| **37042** | F | + | - | - | ASD  (Autism disorder) | Genetic investigation showed MECP2 mutation | Severe social and communication impairment (social withdrawal, no gazing) | Special education clinics |
| **31942** | M | + | - | - | ASD  (PDD-NS +ID +ADHD) | WISC, clinical diagnosis | Language, motor and cognitive delay, no understanding of social rules, no friend, attention deficit, impulsivity and hyperactivity  Has a sibling with ASD | Special class within regular school + ambulatory rehabilitation |
| **30225** | M | - | - | + | ID | Genetic investigation showed phenylketonuria | Severe ID, non-verbal individual | Kindergarten |
| **12658** | F | + | - | - | ID | WIPPSI, CARS | Mild ID, CARS below autism threshold | Kindergarten |
| **35201** | M | + | - | - | ID + ADHD | WIPPSI | Mild ID with anxiety and hyperactivity | Second grade with a helping assistant, a special class with a helping assistant is to be found |
| **5413** | M | + | - | - | ID + ADHD | WIPPSI | Mild ID with hyperactivity | Special class within regular school |
| **20195** | M | + | - | - | ID | Clinical diagnosis | ID, global developmental delay (walking at 22 months), good social contact | Kindergarten |
| **16271** | M |  | + | + | ASD  (Autism disorder) | Specialized clinics, ECA-R | No gazing, social withdrawal, no joint attention or pointing, non-verbal individual  Has a sibling with ASD | Day care hospital |
| **9298** | M | - | + | - | ID+ ADHD | CARS, WIPPSI, ADI-R | Mild ID, CARS below autism threshold, hyperactivity, behavioral impairments | Kindergarten, helping assistant in school to be obtained |
| **15417** | M | - | + | - | ASD  (PDD-NS) | Clinical diagnosis | Motor delay (walking at 21 months), mild social withdrawal, stereotypies, motor coordination impairment, verbal individual with high functioning | 3d grade in a regular school with a helping assistant |
| **38582** | M | - | - | + | ASD  (Autism disorder) | Clinical diagnosis,  PEA, MRI, EEG (seizure) | No language, motor stereotypies, no pointing, use of adult’s hand, no pretending | Not in school yet |
| **14161** | M | - | - | + | ASD  (PDD-NS) | CARS, WIPPSI then WISC High blood lead | Language delay, echolalia and receptive language impairment despite normal IQ, VIQ<PIQ, no gaze, social withdrawal, tantrums, no stereotypies | 3d grade in a regular school with ambulatory special education |
| **13081** | M | - | - | + | ASD  (Asperger) | Clinical diagnosis | No response to name until 19 months, normal language but little delay for oralisation, learned alone to read, perfectionism and still show moment of social withdrawal | 2d grade in a regular school with a helping assistant |
| **13895** | F | + | - | + | ID | Clinical diagnosis | **ID only** | Kindergarten |
| **25415**  ***Negative case*** | ***F*** | *-* | *-* | *-* | ASD  (PDD) | Specialized clinics | Not available | *not specified* |
| ASD: autism spectrum disorder; PDD-NS: pervasive developmental disorder; ID: intellectual disability; ADHD: attention deficit/hyperactivity disorder; WISC: Wechsler intelligence scale for children; WIPPSI: Wechsler preschool and primary scale of intelligence; EEG: electro-encephalogram; MRI: resonance magnetic imaging; CARS: children autism rating scale; ADOS: autism observation schedule; ADI: autism diagnostic interview; VABS: Vineland adaptative behavior scale; ECA-R: *echelle des comportements autistiques révisé*es [scale for autistic behaviors]. | | | | | | | | |
