# Supplementary material for: Infant and dyadic assessment in early community-based screening for autism spectrum disorder with the PREAUT grid
Source: PLoS One. 2017 Dec 7;12(12):e0188831. doi: 10.1371/journal.pone.0188831 (PMC5720624; doi:10.1371/journal.pone.0188831)
Supplement: S3 Table — * Some individuals were positive at several tools. Number of Individuals are rounded to nearest integer. (DOCX) [file pone.0188831.s003.docx]

**S3 Table. Estimation of ASD diagnosis status for (1) infants positive at one screening but lost at FU; (2) infants negative at all screenings and estimated through the random sample.** * Some individuals were positive at several tools. Number of Individuals are rounded to nearest integer.

|  |  |  |  |  |  |  | Method 1 | |  | Method 2 | |  | Estimation (Method 1 – Method 2) | | | | |
| --- | --- | --- | --- | --- | --- | --- | --- | --- | --- | --- | --- | --- | --- | --- | --- | --- | --- |
|  | Screened individuals (N) | Positive individuals at one screening (N) | Positive individuals with FU (N) | Positive individuals with ASD diagnosis at FU (N) | Individuals positive at one screening but lost at FU (N) |  | Raw PPV for ASD | Estimation of ASD in the individuals positive at one screening but lost at FU (N) |  | Adjusted PPV for ASD | Estimation of ASD in the individuals positive at one screening but lost at FU (N) |  | Total true positive (N) | False positives (N) | FN in the subsample of 100 positives (N) | Total False Negatives (N) | True Negatives (N) |
| P4 | 4755 | 22 | 19 | 5 | 3 |  | 26.3 % | 0.79 |  | 20 % | 0.6 |  | 5.79 – 5.6 | 16.21 – 16.4 | 22.21 - 13.4 | 30.39 -21.58 | 4702.61 - 4711.42 |
| P9 | 4530 | 41 | 11 | 4 | 30 |  | 36.4 % | 10.92 |  | 14.3 % | 4.29 |  | 14.92 – 8.29 | 26.08 – 32.71 | 13.08 - 10.71 | 21.26 -18.89 | 4467.74 - 4470.11 |
| C24 | 4835 | 45 | 22 | 6 | 23 |  | 27.3 % | 6.28 |  | 16.7 % | 3.84 |  | 12.28 – 9.84 | 32.72 – 35.16 | 15.72 – 9.16 | 23.90 -17.34 | 4766.1 - 4772.66 |
| All together P4 or P9 or C24* | 4835 | 100 | 45 | 10 | 55 |  |  | 18 |  |  | 9 |  | 28 - 19 | 72 - 81 | 0 - 0 | 8 - 9 | 4727 - 4726 |
