# Supplementary material for: Infant and dyadic assessment in early community-based screening for autism spectrum disorder with the PREAUT grid
Source: PLoS One. 2017 Dec 7;12(12):e0188831. doi: 10.1371/journal.pone.0188831 (PMC5720624; doi:10.1371/journal.pone.0188831)
Supplement: S4 Table — * Some individuals were positive at several tools. Number of Individuals are rounded to nearest integer. (DOCX) [file pone.0188831.s004.docx]

**S4 Table. Estimation of ND (ASD or ID) diagnosis status for (1) infants positive at one screening but lost at FU; (2) infants negative at all screenings and estimated through the random sample.** * Some individuals were positive at several tools. Number of Individuals are rounded to nearest integer.

|  |  |  | |  | |  |  |  | Method 1 | |  | | Method 2 | |  | Estimation (Method 1 – Method 2) | | | | |
| --- | --- | --- | --- | --- | --- | --- | --- | --- | --- | --- | --- | --- | --- | --- | --- | --- | --- | --- | --- | --- |
|  | Screened individuals (N) | | Positive individuals at one screening (N) | | Positive individuals with FU (N) | Positive individuals with ND diagnosis at FU (N) | Individuals positive at one screening but lost at FU (N) |  | Raw PPV for ND | Estimation of ND in the individuals positive at one screening but lost at FU (N) | |  | Adjusted PPV for ND | Estimation of ND in the individuals positive at one screening but lost at FU (N) |  | Total true positive (N) | False positives (N) | FN in the subsample of 100 positives (N) | Total False Negatives (N) | True Negatives (N) |
| P4 | 4755 | | 22 | | 19 | 10 | 3 |  | 52.6 | 1.58 | |  | 46.7 | 1.40 |  | 11.58 -11.40 | 10.42 – 10.60 | 29.42 – 20.60 | 37.60 – 28.78 | 4695.40 – 4704.22 |
| P9 | 4530 | | 41 | | 11 | 5 | 30 |  | 45.4 | 13.62 | |  | 28.6 | 8.58 |  | 18.62 – 13.58 | 22.38 – 27.42 | 22.38 – 18.42 | 30.56 – 26.60 | 4458.44 – 4462.40 |
| C24 | 4835 | | 45 | | 22 | 8 | 23 |  | 36.4 | 8.37 | |  | 22.2 | 5.11 |  | 16.37 – 13.10 | 28.63 – 31.89 | 24.63 – 18 .89 | 32.81 – 27.07 | 4757.19 – 4762.93 |
| All together P4 or P9 or C24* | 4835 | | 100 | | 45 | 17 | 55 |  |  | 24 | |  |  | 15 |  | 41 - 32 | 59 - 68 | 76 - 58 | 8 - 9 | 4726.82 – 4726.82 |
